# Food-specific serum IgG and symptom reduction with a personalized, unrestricted-calorie diet of six weeks in Irritable Bowel Syndrome (IBS)

**DOI:** 10.1186/s12986-020-00528-x

**Published:** 2020-12-01

**Authors:** Mattia Cappelletti, Emiliana Tognon, Linda Vona, Katia Basello, Andrea Costanzi, Michela Carola Speciani, Attilio Francesco Speciani

**Affiliations:** 1 Inflammation Society, 18 Woodlands Park, Bexley, DA52EL UK; 2 SMA Servizi Medici Associati, Via F. Vegezio, 12, 20149 Milan, Italy; 3 GEK Lab - Cryolab University of Rome Tor Vergata, Via Montpellier, 1, 00133 Rome, Italy

**Keywords:** Irritable bowel syndrome, B-cell activating factor, Platelet activating factor, IgG, Nutrition, Diet, Personal food profile

## Abstract

**Background:**

Irritable Bowel Syndrome (IBS) is a widespread disease with variable symptoms that have an important impact on the quality of life. Despite the prevalence of IBS, its etiology and pathophysiology are still to be fully understood, but immune response is known to be involved. In this study, we investigated the variation of two specific cytokines, B-cell activating factor (BAFF) and platelet-activating factor (PAF), the levels of food-specific IgG and the symptom severity, using Irritable Bowel Syndrome—Symptom Severity Score (IBS-SSS), following a personalized and unrestricted-calorie diet.

**Methods:**

We enrolled 30 subjects with diagnosis of IBS, according to Rome-IV criteria, whose inflammatory markers were measured at baseline and after 6 weeks of dietary intervention. The subjects were monitored in a general practice outpatient setting and nutritional advice was offered remotely via two telephone sessions with a nutritionist.

**Results:**

BAFF and PAF values did not differ between baseline and end of study, both in compliant (C) and non-compliant (NC) subjects. IgG levels significantly decreased only in compliant subjects: 37.32 (23.24–93.67) IU/mL; 27.9 (7.56–93.96) IU/mL (*p* = 0.02) and in non-compliant went from 51.83 (13.17–113.1) IU/mL to 44.06 (4.96–255.4) IU/mL (*p* = 0.97, ns). IBS-SSS significantly decreased in both compliant subjects, from 245 (110–480) to 110 (0–140) (*p* < 0.0001), and non compliant subjects, from 250 (155–370) to 100 (7–220) (*p* < 0.0001). Comparing IBS-SSS between week 3 and week 6, only compliant subjects had a significant reduction, from 155 (50–355) to 110 (0–140) (*p* = 0.005), versus non-compliant, from 115 (35–315) to 100 (7–220) (*p* = 0.33, ns).

**Conclusion:**

These findings support the rapid efficacy and suitability of a personalized dietetic intervention with outside consultation in IBS.

*Trial registration*: ClinicalTrials.gov ID NCT04348760 Registered April 15, 2020 (retrospectively registered) https://clinicaltrials.gov/show/NCT04348760

## Introduction

Irritable bowel syndrome (IBS) is a widespread complex clinical condition characterized by chronic abdominal pain or discomfort and altered bowel habits in absence of structural or metabolic abnormalities [1]. Due to the symptoms, IBS has a relevant impact on the quality of life and it is thought to affect around 12% of the global population [2]. Despite its prevalence and the disease burden, its pathophysiology and all of the underlying mechanisms remain largely unclear, partly because of its multifactorial etiology. It is known that genetic and environmental factors play a role in the development of IBS [3, 4]. Recently, there has been mounting evidence of a key role played by immunity and inflammation in both the genesis and persistence of the disease [5–7]. Moreover, it has been demonstrated that an altered systemic immune response is active in IBS patients, with the release of pro-inflammatory cytokines like B-cell activating factor (BAFF), Interleukin 1B (IL-1B), Tumor necrosis factor alpha (TNF-a), Interleukin 6 (IL-6) and Interleukin 8 (IL-8) [8–12]. Mastcells and their mediators, like Platelet-activating factor (PAF), also play a key role in this setting, inducing degranulation and chemical mediators release through the interaction between immune complexes of food-specific IgG and complement activation [6, 7, 12]. Once activated, mastcells are able to release a wide variety of mediators increasing the excitability of both intrinsic enteric neurons, which regulate motility and secretion, and afferent extrinsic neurons which provide also pain signaling to the central nervous system [13].

Since 2007, numerous studies have documented the relationship between the increase in specific cytokines like BAFF and PAF and the excessive or repeated intake of specific food groups [9, 14–16]. More specifically, BAFF is secreted by intestinal mucosa when certain food antigens interact with toll-like receptors (TLR), determining an inflammatory response [17, 18]. Moreover, Kang in 2016 documented that IgG are able to bind with antigens that pass through the intestinal mucosa, forming immune complexes. The presence of these complexes activates B-cell response and, in the presence of normal to high BAFF concentrations, the production of antibodies and the release of pro-inflammatory cytokines, including BAFF itself [12]. This demonstration may represent the link between the presence of food-specific IgG and the activation of the adaptive immune response.

Relying on these studies, it has been possible to understand how the repeated daily intake of certain food groups is able, on an individual basis, to trigger the increase of chronic low-grade inflammation and then, in a direct or indirect way, the insurgence or maintenance of chronic inflammatory diseases like IBS. It is also known that IBS patients tend to have altered levels of food-specific IgG [19].

Measuring the levels of food-specific IgG, whose increase is proportional with the intake of the specific foods [16], we are then able to assess personal eating habits and food antigen exposure, defining a personalized nutritional approach with the aim of balancing such exposure and then reducing systemic inflammation. The choice of BAFF and PAF as elective cytokines was based on previous preliminary studies which determined their similar behavior and their key role in food inflammation and non-IgE mediated food reactions [9, 20].

The aim of this study was to determine the short-term variation of BAFF, PAF and food-specific IgG following a personalized dietary approach. The monitoring of IBS symptoms, using a validated score, associated with the nutritional change was also a key part of the study.

## Materials and methods

### Study design and subjects

We conducted this prospective interventional study enrolling 30 subjects, male and female, between age 18 and 50, with IBS, at 6 general practitioners offices in Genova, Italy. All the subjects had to meet Rome-IV criteria for the diagnosis of IBS, consisting in recurrent abdominal pain at least 1 day/wk, on average, in the last 3 months, with symptoms beginning at least in the last 6 months, associated with two or more of the following alterations: (1) related to defecation; (2) associated with a change in frequency of stool; and (3) associated with a change in appearance of stools [21]. Major exclusion criteria were: known inflammatory bowel disease (e.g. Crohn, Ulcerative Colitis), alcohol abuse, eating behavior disorders, diabetes, coeliac disease, chronic therapy with drugs known to alter intestinal motility or adsorption (e.g. thyroxine, PPIs).

Anthropometric measurements (weight, height, circumference at waist, abdomen and hips) and an IBS symptom assessment (Irritable Bowel Syndrome Symptom Severity Score, IBS-SSS) [22] was obtained by the general practitioner at enrollment, after 3 weeks and after 6 weeks (end of study, EOS). At enrollment and after 6 weeks we obtained a blood sample via finger puncture for the measurement of BAFF, PAF and food-specific IgG. After enrollment and after each visit performed by the general practitioner (week 3 and EOS), a telephone consultation with a specialized nutritionist was offered to explain the dietary intervention and to evaluate compliance.

### Dietary intervention

The proposed nutritional pattern agreed with the Healthy Eating Plate proposed by Harvard School of Public Health [23]. The daily total energy intake was distributed across three main meals (breakfast, lunch, and dinner). Based on the food-specific IgG measurement and relative distribution, a personalized food profile was created for each subject identifying 1 to 3 relevant food groups/nutritional clusters [24]. Subjects were then instructed to avoid the foods highlighted in their personal food profile in certain days of the week, and to assume them only in 7 of the 21 meals of the week (two full days and another meal of choice). In this way each subject was instructed to restrict the food groups (highlighted in their own personal results) that were more frequently consumed in their own usual diet (i.e. gluten, dairy, nickel), forcing the consumption of aliments from different food groups and thus increasing food variability. The adherence to this plan, achieving at least a whole day of avoidance of the food groups in their personal profile was the determining factor in IBS symptom and food-related IgG reduction.

No calorie restriction was imposed in the diet. Compliance was assessed via the combined analysis of a 3-day food diary compiled by each subject and 48-h food recollection obtained during the telephone consultations with a specialized nutritionist at week 3 and week 6. Given the instructions to avoid the food groups in almost 5 days of the week (with assumption permitted only in two full days plus another meal of choice), subjects were considered non-compliant (NC) if they failed to achieve at least one full day of abstinence from the food groups in the combined assessment.

### Laboratory studies

B-cell Activating factor (BAFF), and Human Platelet Activating Factor (PAF) were measured via commercial ELISA kits (Human BAFF/BLyS/TNFSF13B Immunoassay Quantikine® ELISA, Catalog Number PDBLYS0B, R&D Systems Inc, Minneapolis, MN, USA; Human Platelet Activating Factor ELISA Kit, Catalog Number E-EL-H2199, Elabscience Houston, TX, USA, respectively) using the Biomek 4000 ELISA microplate liquid reagent dispensing automation tool (Beckman Coulter, Brea, CA, USA) and the EL405LS ELISA microplate automated washing system (BioTek Instruments, Winooski, VT, USA). The absorbance of each well was read at a wavelength of 450 nm with a Multiskan FC plate reader (Thermo Scientific, Waltham, MA, USA). The average zero standard optical density was subtracted from all absorbances, and a standard curve was generated by using a four-parameter logistic (4-PL) curve fit. The concentration in the test sample was calculated through interpolation along the standard curve by multiplying the result by the dilution factor.

Serum IgG responses to food antigens is evaluated by sensitive and specific ELISA (enzyme-linked immunosorbent assay) test using the Automatic Workstation Biomek i7 (Beckman Coulter, Brea, CA, USA). Food antigens and reference antigen for standards and controls are bound to the plate. Diluted patient serum or standards are pipetted into the wells of the microtiter plate. A binding between the IgG antibodies of the serum and the immobilized antigens takes place. After one hour incubation at 37 °C, the plate is rinsed with diluted wash solution in order to remove unbound material. Then Goat anti-human-IgG-HRP conjugate (Catalog Number 62-8420, Thermo Fisher Scientific, Rockford, IL, USA) is added and incubated for 30 min at 37 °C. After a further automatic washing step, the substrate 3,3′,5,5′-Tetramethylbenzidine (TMB) solution (Surmodics IVD Inc., Eden Praire, MN, USA) is pipetted and incubated for 20 min at 37 °C, inducing the development of a blue colour in the wells. The colour development is modified by the addition of a stop solution (sulfuric acid 2 N). The resulting yellow colour is measured spectrophotometrically at the wavelength of 450 nm. The concentration of the IgG antibodies is directly proportional to the intensity of the colour. The standards of the IgG ELISA are commercially available human IgG defined and expressed in IU/mL (cat. n. I4506, Sigma Aldrich, St. Louis, MO, USA). This results in an exact and reproducible quantitative evaluation. Consequently for a given patient follow-up controls become possible. For a quantitative evaluation the absorbances of the standards are graphically drawn against their concentrations. From the resulting reference curve the concentration values for each patient sample can then be extracted in relation to their absorbances. The lower limit of detection is 0.1 IU/mL with the intra- and inter-assay reproducibility of 4.9% and 6.9%, respectively.

### Statistical analysis

Statistical analysis of the data was performed using GraphPad Prism 8 for macOS (GraphPad Software, San Diego, CA, USA. Version 8.3.0 (328), 16 October 2019). The data is presented as median and range. The medians were compared using the Mann–Whitney test. A *p* value < 0.05 was used as the limit of statistical significance.

## Results

We enrolled 30 subjects, 12 males and 18 females, whose mean age was 41 (23–50) years. All subjects completed the 6-week period of study, but only 13 achieved a sufficient compliance with the proposed dietary modification. Compliant (C) and non-compliant (NC) subjects did not show any significant difference at baseline, so the results are split between them to show similarities and differences.

In both groups BAFF did not differ between baseline and end of study: BAFF (C) went from 0.45 (0.2–0.6) ng/mL to 0.43 (0.3–0.63) ng/mL; BAFF (NC) went from 0.38 (0.33–0.95) ng/mL to 0.40 (0.14–0.89) ng/mL. Likewise, PAF levels did not show significant differences between C and NC subjects: PAF (C) went from 4.04 (0–21) ng/mL to 4.68 (0–39) ng/mL; PAF (NC) went from 6.07 (0.81–21.8) ng/mL to 7.64 (0.53–30.5) ng/mL. Food-specific IgG levels significantly decreased only in compliant subjects going from 37.32 (23.24–93.67) IU/mL to 27.9 (7.56–93.96) IU/mL (*p* = 0.02) and in non-compliant went from 51.83 (13.17–113.1) IU/mL to 44.06 (4.96–255.4) IU/mL (*p* = 0.97, ns). IBS-SSS significantly decreased in both groups: in compliant went from 245 (110–480) to 110 (0–140) (*p* < 0.0001) and in non-compliant subjects from 250 (155–370) to 100 (7–220) (*p* < 0.0001). A further analysis, comparing IBS-SSS between week 3 and week 6, shows that compliant subjects still had a significant reduction, going from 155 (50–355) to 110 (0–140) (*p* = 0.005), while non-compliant ones did not have the same trend, going from 115 (35–315) to 100 (7–220) (*p* = 0.33, ns).

In Tables 1 and 2 are shown, respectively, compliant and non-compliant analytical data.

**Table 1:** Analytical data of compliant subjects (C). BAFF and PAF did not differ between baseline and week 6. A statistically significant reduction in food-specific IgG levels can be seen only in this group. IBS-SSS also had a significant reduction

	Baseline	Week 6	*p*
BAFF (ng/mL)	0.45 (0.2–0.6)	0.43 (0.3–0.63)	ns
PAF (ng/mL)	4.04 (0–21)	4.68 (0–39)	ns
IgG (IU/mL)	37.32 (23.24–93.67)	27.9 (7.56–93.96)	0.02
IBS-SSS (pt)	245 (110–480)	110 (0–140)	< 0.0001

**Table 2:** Analytical data of non-compliant subjects (NC). BAFF and PAF did not differ between baseline and week 6. In NC subjects there was no statistically significant reduction in food-specific IgG levels. IBS-SSS had a significant reduction

	Baseline	Week 6	*p*
BAFF (ng/mL)	0.38 (0.33–0.95)	0.40 (0.14–0.89)	ns
PAF (ng/mL)	6.07 (0.81–21.8)	7.64 (0.53–30.5)	ns
IgG (IU/mL)	51.83 (13.17–113.1)	44.06 (4.96–255.4)	ns
IBS-SSS (pt)	250 (155–370)	100 (7–220)	< 0.0001

## Discussion

After enrollment and the beginning of nutritional intervention, a rapid reduction in the symptoms in the vast majority of subjects was observed. This symptom-reduction trend was confirmed both at week 3 and EOS and supports the efficacy of a balanced diet and the maintenance of food variability in the weekly diet.

The relative stability of BAFF and PAF, despite the symptom reduction, may indicate that a clinical response precedes a biochemical one, and in a multifactorial disease like IBS may also indicate that even more pathways are implicated in the genesis of clinical symptoms.

**Fig. 1:**
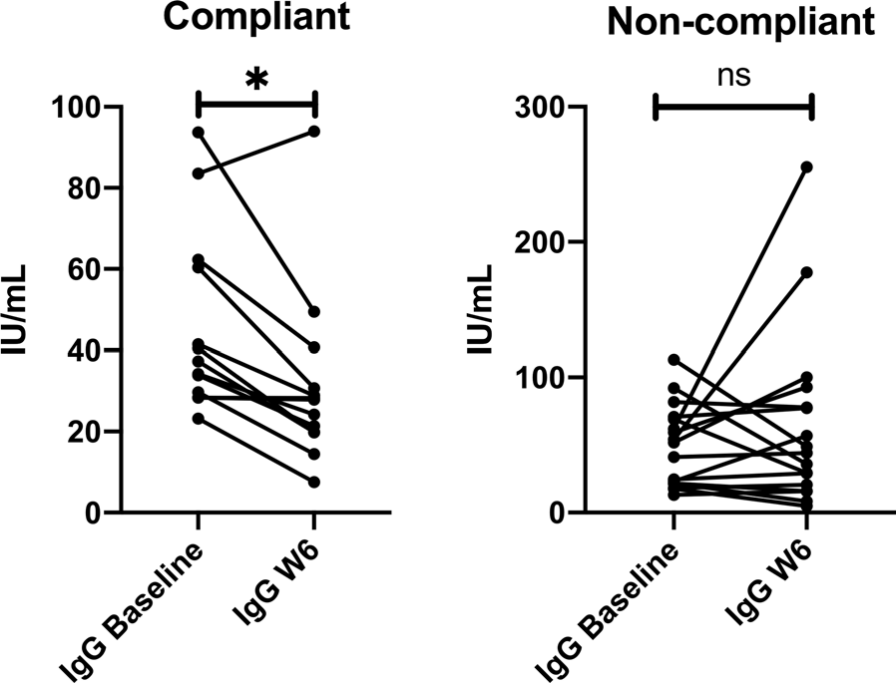
IgG trend in compliant and non-compliant subjects between baseline and week 6. In C subjects there was a statistically significant reduction between baseline and week 6, with a clearly visible reduction trend. In NC subjects no significant reduction was observed, and many subjects faced an increase in food-specific IgG levels

The significant reduction of food-specific IgG, which occurred only in the compliant group, as in Fig. 1, supports the use of a personal food profile in guiding an individualized nutritional approach to modulate food antigen exposure and food-specific IgG levels.

**Fig. 2:**
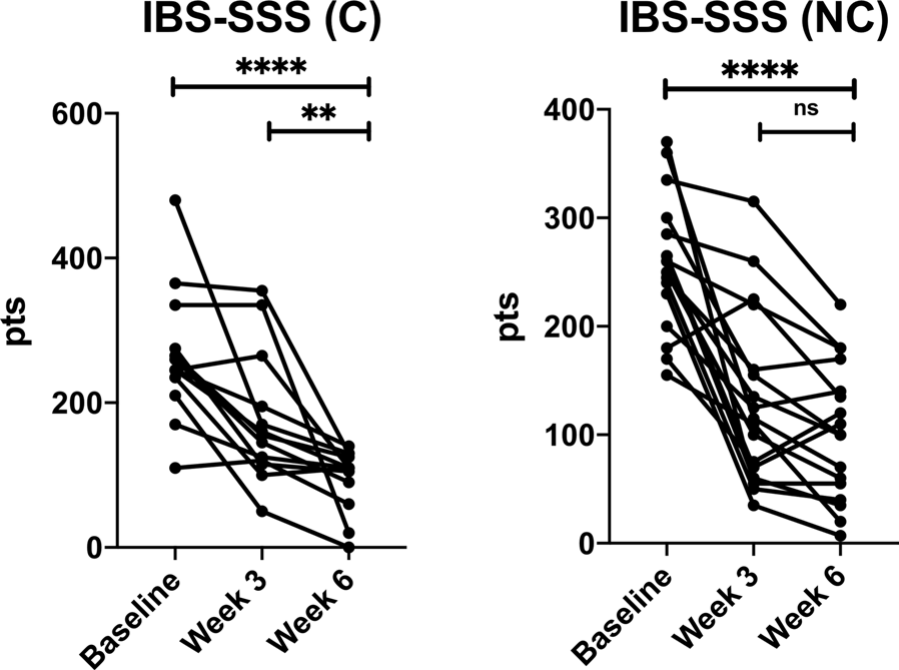
Linear trend of IBS-SSS in compliant and non-compliant subjects. In C subjects the reduction trend was maintained and significant both between baseline and week 6, and between week 3 and week 6. In NC subjects the reduction was significant only between baseline and week 6, but between week 3 and week 6 there was no difference. In fact, a “symptom rebound” could be seen in some subjects

The reduction of symptom severity was significant in both groups, but this may be due to the fact that nearly every subject in the study had some level of modification of their dietary habits and, given the short duration of the study, we have observed the initial positive effect of both optimal and “suboptimal” diets. Going deeper into the analysis of this aspect, Fig. 2 shows that in the compliant group all subjects show a constant trend in the reduction of the severity of symptoms at EOS, while in the non-compliant group some subjects show a rebound between week 3 and week 6, as if the lack of adherence was leading to a reappearance of symptoms. In support of this hypothesis, comparing IBS-SSS between week 3 and week 6, the reduction trend is statistically significant only in compliant subjects while in non-compliant ones the reduction is negligible. Probably a longer period of observation could have better highlighted this aspect. This trend can also be representative of one of the biggest obstacles in dietary interventions: long-term compliance. In fact, most of the subjects modified their usual diets in the first couple of weeks (which could explain the week 3 results) but then returned to their usual diets in the following ones, losing food variability and failing to maintain at least a full day of avoidance of the food groups in their personal profile. This lack of adherence appears to be the determining factor of constant reduction trend in IBS-SSS.

The use of outside consultation, in this case two phone appointments, in conjunction with a personalized dietary plan has proved to be a viable way of delivering effective care for patients with IBS, opening the possibility for treatment even in settings of limited time and resources.

**Table:** Summary table

Background/rationale	IBS is a widespread disease with a pathophysiology largely unknown. We investigated the role of an easy to deliver personalized dietary intervention in the reduction of inflammatory markers and IBS symptom severity
Methodology	30 subjects with diagnosis of IBS (according to Rome-IV criteria) whose inflammatory markers and food specific IgG levels were measured at baseline and after 6 weeks of dietary intervention. IBS Symptom severity was monitored at baseline and after 3 and 6 weeks Monitoring was provided in a general practice outpatient setting and nutritional advice was offered remotely via two telephone sessions with a certified nutritionist
Results	BAFF and PAF values did not differ between baseline and end of study, both in compliant (C) and non-compliant (NC) subjects. Food-specific IgG levels significantly decreased only in compliant subjects. IBS-SSS significantly decreased in both groups. Comparing IBS-SSS between week 3 and week 6, only compliant subjects had a significant reduction
What is known about the subject	Chronic systemic Inflammation and dietary habits have been variously linked to IBS symptoms
What does this study add	The identification of a personal food profile and a personalized dietary intervention was able to rapidly reduce both IBS symptoms and food-specific IgG levels in IBS patients, thus providing a simple and effective way of treatment for these patients
Future perspectives	Larger studies with longer follow-up are needed to further evaluate this approach

### Limitations

The population of this study is small and the results need confirmation in larger settings, but these findings should encourage the promotion of further studies. The observation period is relatively small and this may explain the lack of biochemical response regarding BAFF and PAF, and the reduction of IBS-SSS in both compliant and non-compliant subjects. On the other hand, the study showed the rapid efficacy of this approach.

## Conclusion

The aim of this study was to determine the short-term variation of BAFF, PAF, food-specific IgG and severity of symptoms following a personalized dietary approach in IBS patients. The results showed the rapid efficacy of an individualized dietary approach on IBS symptoms and a prompt reduction of food-specific IgG. This could suggest a more widespread use of this approach, also including the use of remote counseling that offers a unique flexibility and a more oculate use of resources, and should promote further research in this treatment setting.

## Data Availability

The datasets used and/or analyzed during the current study are available from the corresponding author on reasonable request.

## Abbreviations

IBS: Irritable bowel syndrome
BAFF: B-Cell Activating Factor
PAF: Platelet Activating Factor
IBS-SSS: Irritable Bowel Syndrome-Symptom Severity Score
C: Compliant
NC: Non compliant
ELISA: Enzyme-linked immunosorbent assay
EOS: End of study
